# Integrative Analysis of MicroRNA and Gene Interactions for Revealing Candidate Signatures in Prostate Cancer

**DOI:** 10.3389/fgene.2020.00176

**Published:** 2020-02-27

**Authors:** Jingchao Wei, Yinghao Yin, Qiancheng Deng, Jun Zhou, Yong Wang, Guangming Yin, Jianfu Yang, Yuxin Tang

**Affiliations:** ^1^Department of Urology, The Third Xiangya Hospital of Central South University, Changsha, China; ^2^Department of Dermatology, The Second Xiangya Hospital of Central South University, Changsha, China; ^3^Department of Urology, Hunan Provincial People’s Hospital, Changsha, China

**Keywords:** prostate cancer, signature, microRNA, WGCNA, TCGA

## Abstract

MicroRNA (miRNA)–gene interactions are well-recognized as involved in the progression of almost all cancer types including prostate cancer, which is one of the most common cancers in men. This study explored the significantly dysregulated genes and miRNAs and elucidated the potential miRNA–gene regulatory network in prostate cancer. Integrative analysis of prostate cancer and normal prostate transcriptomic data in The Cancer Genome Atlas dataset was conducted using both differential expression analysis and weighted correlation network analysis (WGCNA). Thirteen genes (*RRM2, ORC6, CDC45, CDKN2A, E2F2, MYBL2, CCNB2, PLK1, FOXM1, CDC25C, PKMYT1, GTSE1, and CDC20*) were potentially correlated with prostate cancer based on functional enrichment analyses. MiRNAs targeting these genes were predicted and eight miRNAs were intersections between those miRNAs and the hub miRNAs obtained from miRNA WGCNA analysis. Three genes (*E2F2, RRM2, and PKMYT1*) and four miRNAs (*hsa-mir-17-5p, hsa-mir-20a-5p, hsa-mir-92a-3p, and hsa-mir-93-5p*) were key factors according to the interaction network. *RRM2* and *PKMYT1* were significantly related to survival. These findings partially elucidated the dysregulation of gene expressions in prostate cancer. Efficient manipulations of the miRNA–gene interactions in prostate cancer may be exploited as promising therapeutics.

## Introduction

Prostate cancer ranks as one of the most common malignancies in men (Attard et al., 2016). High-risk prostate cancer is aggressive and can lead to poor outcomes at advanced stages and metastasis (Litwin and Tan, 2017). A tremendous number of investigations have been conducted with the goal of determining the mechanisms underlying the oncogenesis and development of prostate cancer. Significant findings including androgen receptor mechanism have been well-established (Culig and Santer, 2014). However, the detailed underlying mechanism is still far from being clear. To improve the management of prostate cancer, the identification of novel signatures is of vital importance for the prediction of prognosis and targeted therapy of prostate cancer patients.

MicroRNAs (miRNAs) are small, non-protein-coding RNA (usually 22 nucleotides) molecules, which function in RNA silencing and post-transcriptional regulation of target gene expression (Rupaimoole and Slack, 2017). Accumulating evidence has shown that miRNAs are involved in nearly all cancer types, acting as tumor oncogenes or suppressors and taking part in cell proliferation, differentiation, and metastasis (Winbanks et al., 2014; Vorvis et al., 2016). It is well-established that a large number of specific miRNAs are involved in the carcinogenesis and development of tumors by regulating the expressions of their targeted mRNAs (Inui et al., 2010). With advancements in biological and clinical research, miRNA–mRNA interactions have been widely demonstrated to regulate the complicated molecular mechanisms underlying oncogenesis, development, invasion, and metastasis of tumors (Duell et al., 2017; Han et al., 2017). The Cancer Genome Atlas (TCGA) database has stored numerous genomic and gene expression profiles for various types of tumors. Many genes and signaling pathways correlated with cancer have been predicted based on analyses of these data, which have provided valuable guidance for subsequent molecular biology validation (The TCGA Legacy, 2018).

This study investigated the differentially expressed genes (DEGs) and hub miRNAs in prostate cancer and screened signatures of prostate cancer. Integrative analysis was performed using gene and miRNA expression data of prostate cancer downloaded from TCGA database to identify DEGs and miRNAs in prostate cancer when compared with adjacent normal tissue samples. Functional enrichment analyses were subsequently conducted. Then the mRNA–miRNA interaction network was constructed. Three genes and four miRNAs were found to be hub factors of prostate cancer and the prognostic significance was investigated.

## Materials and Methods

### Analysis of Differentially Expressed Genes Between Prostate Cancer and Normal Samples in TCGA Prostate Adenocarcinoma (PRAD) Datasets

Transcriptome raw count data of TCGA PRAD were obtained from GDC Data Portal using R TCGAbiolinks package (Colaprico et al., 2016). Clinical data of TCGA PRAD subjects were obtained from UCSC XENA browser.^1^ Samples of primary solid tumor and solid normal tissue were picked out for analysis, and paraffin embedded samples as well as metastatic samples were removed. Ensemble identifiers were annotated with gene names using R Annotables package (genome version GRCh38). Identifiers with no annotations were removed. For duplicated identifiers corresponding to one gene name, the average of all count values was calculated and rounded to be taken as the count of that gene. Analysis of DEGs between prostate cancer and normal tissues were performed using R DESeq2 package (Love et al., 2014). The phenotype of tumor or normal sample was used as grouping variable and standard differential expression analysis was conducted. The normalized expression matrix was obtained through variance stabilizing transformation of raw count data. As the DEGs would be taken as input in the subsequent analysis, a less stringent criterion was used to identify DEGs: adjusted *p*-value < 0.05 and absolute value of fold change ≥ 2. Principal component analysis (PCA) was performed based on the normalized expression data, using prcomp function in R. Pheatmap and ggplot2 packages were used to plot gene expression boxplot, volcano plot and heatmap.

### Weighted Correlation Network Analysis of DEGs and the Identification of Modules Associated With Prostate Cancer

The DEGs obtained by DESeq2 were used to perform weighted correlation network analysis (WGCNA) to get the interesting gene modules based on the gene expressions and patient traits using R WGCNA package (Langfelder and Horvath, 2008). Before the one-step network construction and module detection, a soft thresholding power value was calculated to produce a scale-free network topology. Then one-step network construction and detection of consensus modules were executed. The network type was set as signed. Correlations between clinical traits (tumoral or normal) and each module were calculated and preliminary Kyoto Encyclopedia of Genes and Genomes (KEGG) analyses were conducted for genes in several top trait-correlated modules to determine the module that made the most sense. Black module was speculated to be a candidate tumor-driving module since preliminary KEGG analyses revealed some interesting pathways potentially related with tumor biology.

### Functional Enrichment Analyses of Genes in Significantly Correlated Modules

To examine the biological functions of the genes in black modules, we performed functional enrichment analyses using KEGG, Gene Ontology (GO), and REACTOME Pathway databases. KEGG pathway enrichment analysis was conducted using the R package clusterProfiler (Yu et al., 2012). Genes in the black module were annotated with R package Annotables and all DEGs obtained by DESeq2 were used as background. Twelve pathways were significantly enriched (adjusted *p*-value < 0.05), and genes in five interesting pathways among the 12 pathways were picked out for subsequent GO biological process analysis, which was performed using g:GOST tool in gProfiler.^2^ gProfiler is a web server for functional enrichment analysis and conversions of gene lists. The tool g:GOSt in gProfiler performs functional enrichment analysis on input gene list (Raudvere et al., 2019). Among all significantly enriched biological processes, the top 20 were selected for investigation, and the associated genes in all 20 interesting terms were used for subsequent REACTOME Pathway enrichment analysis using the REACTOME Pathway analysis tool.^3^ Among all significantly enriched REACTOME pathways, we studied the top 20 and identified 13 genes in nine pathways that were potentially prostate cancer gene drivers. Enrichplot, ggplot2, and pheatmap packages were used for plotting.

### Weighted Correlation Network Analysis of miRNAs and Identification of Hub miRNAs Associated With Prostate Cancer

Normalized transcriptome data of miRNA [log2(total_RPM + 1) transformed] in prostate cancer were obtained from UCSC XENA.^1^ Likewise, samples of primary solid tumor and solid normal tissue were selected for the analysis, and paraffin embedded samples and metastatic samples were removed. MiRNA identifiers were annotated with detailed names using the R MiRBaseConverter package (Xu et al., 2018) (target version V22). Identifiers with no annotations were removed. For duplicate identifiers corresponding to one miRNA name, the average of all values was calculated to represent the expression value for that miRNA. Then the processed miRNA expression data were used to perform WGCNA to obtain interesting miRNA modules correlated with patient traits (tumoral or normal). The pattern for constructing one-step network and detecting consensus miRNA modules was similar to that of the aforementioned genes. Correlations between clinical traits and each module were calculated and the turquoise module was the most correlative module with prostate cancer. Then top-ranked miRNAs that had maximum connectivity with other miRNAs were determined by the signedKME function in the turquoise module.

### Construction of the Gene–miRNA Interaction Network and Identification of Prostate Cancer Signatures

The MiRNet tool,^4^ which is an integrated platform linking miRNAs, was used to collect all miRNAs that were predicted or validated to target the 13 candidate gene drivers. The miRNA–gene interaction data of miRNet were collected from miRTarBase v7.0, TarBase v7.0 and miRecords, catalogs that predict and validate miRNA–gene interactions (Xiao et al., 2009; Vlachos et al., 2015; Chou et al., 2018). The validated miRNA–gene interactions are manually curated and experimentally validated, and the predicted miRNA–gene interactions are produced by established miRNA target prediction programs. MiRNAs targeting at least two genes were screened to identify intersections with the hub miRNAs obtained by WGCNA analysis. Gene–miRNA interactions were plotted using Cytoscape software (Shannon et al., 2003).

### Kaplan–Meier Survival Analyses and Receiver Operating Characteristic Curves of the Three Gene Signatures

To investigate the clinical significance of the three gene signatures, we performed Kaplan–Meier survival analyses and receiver operating characteristic (ROC) curves for survival. Survival data of TCGA Prostate Adenocarcinoma were obtained from the UCSC XENA platform, and the gene expression data used in this section were the aforementioned normalized expression matrix obtained by DESeq2 package. Kaplan–Meier survival analyses were conducted utilizing survfit function in the R Survival package. Survival ROC curve analyses for survival prediction were performed using roc function in the R proc package based on the miRNA expression data obtained from UCSC XENA (Robin et al., 2011). Area under curve (AUC) was calculated to measure how well the model could distinguish between survival and non-survival classes.

### Validation of Expression of Four miRNA Signatures in Prostate Cancer Using Gene Expression Omnibus Datasets

To analyze the expression of four miRNA signatures in current high-throughput projects on prostate cancer, we used dbDEMC (version 2.0^5^), which is an integrated database designed to store and display differentially expressed miRNAs in human cancers detected by high-throughput methods (Yang et al., 2017). R limma package was used to screen the differentially expressed miRNAs from cancer compared with the normal state (Ritchie et al., 2015). Fold changes and *p*-values were plotted using ggplot2 package.

### Data Processing and Statistical Analysis

Data sources were mentioned above and R Programming software was obtained from the R official website (version 3.6.0^6^). Rstudio software was obtained from the official website.^7^ *p* < 0.05 was considered statistically significant.

## Results

### Analysis of Differentially Expressed Genes (DEGs) Between Prostate Cancer and Normal Samples in TCGA Datasets

**FIGURE 1:**
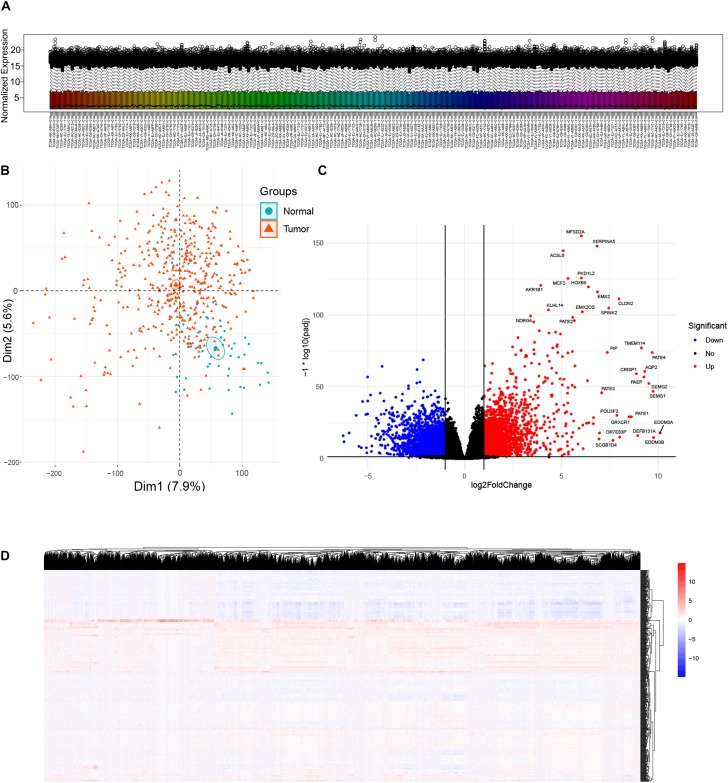
Analysis of differentially expressed genes (DEGs) between prostate cancer and normal samples in TCGA Prostate Adenocarcinoma (PRAD) datasets. **(A)** Box plot showing expression distributions for normalized data for each sample. The boxes are randomly colored with rainbow colors. Normalization method: Variance Stabilizing Transformation/DESeq2 package. **(B)** Principal component analysis (PCA) plot of the mRNA expression data based on the top two principal components that characterizes the trends exhibited by the expression profiles of tumor and normal tissues, respectively. Each dot represents a sample and each color represents the type of the sample. **(C)** Volcano plot showing DEGs between tumor and normal tissues. X-axis represents log2 fold change and Y-axis represents –log10 (adjusted *p-*value). Red dots represent upregulated DEGs (*n* = 3157) and blue dots represent downregulated DEGs (*n* = 3367) in prostate cancer. DEGs screening cutoff: fold change =2 and adjusted *p*-value < 0.05. **(D)** Heat map displaying hierarchical clustering of DEGs (*n* = 6524). Red denotes increased gene expression levels and blue denotes decreased levels.

A total of 481 primary solid tumor samples and 51 solid normal samples with clinical data were screened after paraffin embedded samples and metastatic samples were removed. The DESeq2 package estimates variance-mean dependence in count data of sequencing assays and tests for differential expression based on a model using the negative binomial distribution. Since raw count data are not normally distributed, it was necessary to transform the data to a normalized scale. A normalized gene expression matrix was obtained using variance stabilizing transformation function of the DESeq2 package. Box plot showed expression distributions for normalized data for each sample (Figure 1A). Overall, the expression distributions of raw normalized-intensities were not identical but were similar, as no sample was that different from others. The PCA plot provided insights into the similarities among samples and indicated the quality of the expression data, which can effectively discriminate low-quality samples. In this study, PCA plot based on the first two components, as they described the largest variability, showed tumor and normal samples were roughly distinguished (Figure 1B). A total of 6524 DEGs (both upregulated and downregulated genes in cancer samples compared to normal sample) were identified according to the criterion of adjusted *p*-value < 0.05 and absolute fold change ≥ 2 (Figure 1C). Heatmap showed the DEG expressions in all samples (Figure 1D).

### WGCNA of DEGs

**FIGURE 2:**
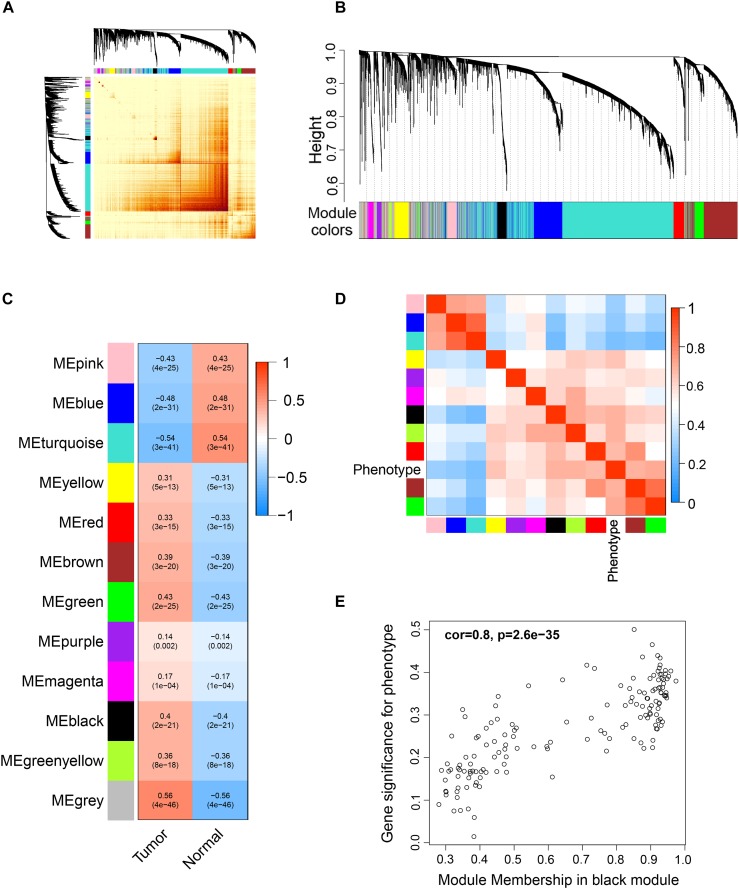
Weighted correlation network analysis (WGCNA) of DEGs. **(A)** Topological overlap matrix (TOM) plot showing topological overlap in the gene network of the DEGs obtained by DESeq2. Each row and column denote a gene and the depth of the red color is positively correlated with the strength of the correlation between the pairs of modules on a linear scale. Blocks of darker colors along the diagonal correspond to the modules. The gene dendrogram and module assignment are shown along the left and top. **(B)** Clustering dendrograms for the DEGs with dissimilarity based on the topological overlap together with the assigned module colors. Eleven co-expression modules were constructed with various colors. The leaves of the tree denote the DEGs, and the height reflects the closeness of individual genes. **(C)** Module-trait relationships. Each row corresponds to a co-expression module, each column corresponds to a trait, and each cell contains the corresponding correlation coefficient and *p*-value. The table is color-coded according to the color legend on the right. **(D)** Heatmap plot of the adjacencies of modules. Red represents high adjacency (positive correlation) and blue represents low adjacency (negative correlation). **(E)** A scatter plot of correlation between black module eigengene and tumor phenotype. Correlation coefficient and *p*-value is indicated in the plot.

**FIGURE 3:**
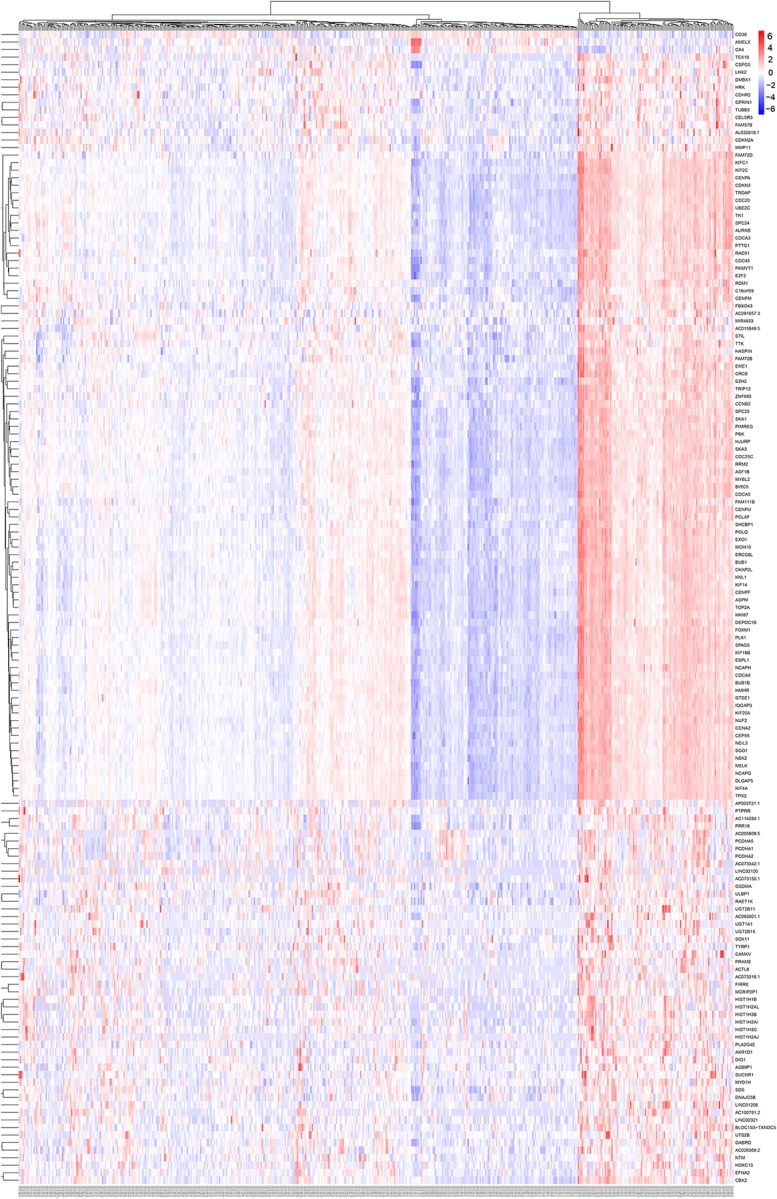
Heatmap and cluster analysis (Euclidean distance) of the genes in black module obtained by WGCNA analysis of DEGs. Rows correspond to genes and columns correspond to samples. Red and blue colors indicate different extents of high or low expressions.

Differentially expressed genes were used for WGCNA analysis. WGCNA is an unsupervised analysis method that clusters genes based on their expression profiles. It is widely used to study the relationships between co-expression modules (clusters) and the relationships between modules and external sample traits (Langfelder and Horvath, 2008). In our study, value 4 was chosen as the soft thresholding power value because it produced a higher similarity with a scale-free network and contributed to gene clustering. A one-step network was constructed using blockwiseModules function with the parameters: of signed Topological Overlap Matrix type and Pearson’s correlation coefficient (Figure 2A). Clustering dendrograms for DEGs with dissimilarity and assigned module colors were plotted (Figure 2B), and 11 modules with a minimum module size of 30 genes were obtained. The correlations between each module and trait (prostate cancer or normal prostate) were calculated (Figure 2C), and connection strengths (adjacencies) of the modules and traits are shown (Figure 2D). Red indicates high adjacency (positive correlation) and blue indicates low adjacency (negative correlation). Here, we identified some genes that were strongly positively correlated with prostate cancer. Among all of the modules, the green and black modules had the highest correlations with prostate cancer (excluding gray module), and preliminary KEGG pathway enrichment analyses were performed to evaluate their biological significance. Several interesting pathways correlated with cancer were enriched by black modules, whereas analysis of the green module did not yield significant results. The black module eigengenes were highly correlated with prostate cancer (Figure 2E). Thus black module was speculated to be a potential prostate cancer-related module and was utilized for subsequent analyses. Heatmap showed the expressions of all genes in the black module (Figure 3).

### Functional Enrichment Analysis

**FIGURE 4:**
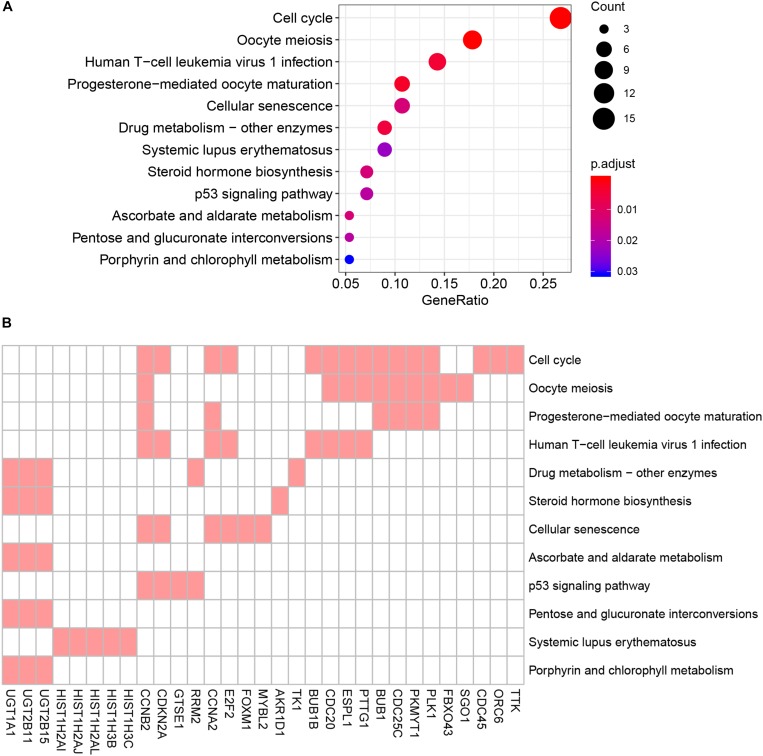
Kyoto Encyclopedia of Genes and Genomes (KEGG) pathway analysis of genes in black module. **(A)** Advanced bubble chart indicates enrichment of 31 genes in 12 KEGG signaling pathways. Y-axis labels correspond to pathways, and X-axis labels correspond to gene ratio (number of input genes in this pathway/number of all genes in this pathway). Color and size of the bubble correspond to enrichment significance and amount of input genes enriched in pathway, respectively. **(B)** Heatmap of the 31 genes enriched in significant KEGG pathways. Red boxes mean that the corresponding gene (X-axis) is enriched in the corresponding pathway (Y-axis).

**FIGURE 5:**
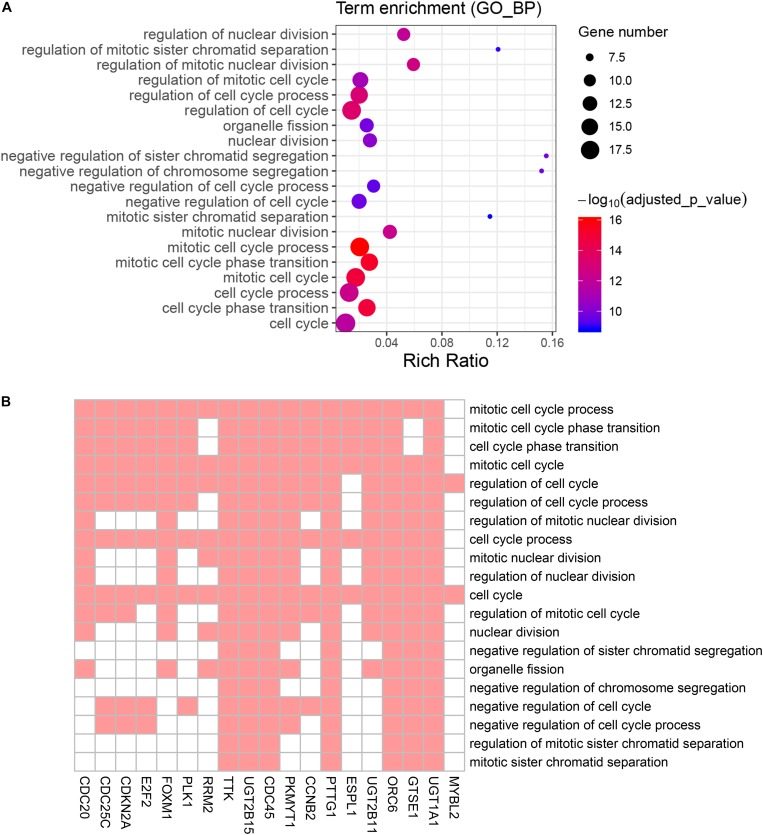
Gene Ontology (GO) biological process enrichment analysis of the genes screened by KEGG pathway analysis. **(A)** Advanced bubble chart shows enrichment of 19 genes in top 20 (based on adjusted *p*-value) enriched GO biological processes. Y-axis labels correspond to biological processes, and X-axis labels correspond to rich ratio (number of input genes in this biological process/number of all genes in this biological process). Color and size of the bubble correspond to enrichment significance and amount of input genes enriched in biological process, respectively. **(B)** Heatmap of the 19 genes enriched in significant GO biological processes. Red boxes mean that the corresponding gene (X-axis) is enriched in the corresponding biological process (Y-axis).

**FIGURE 6:**
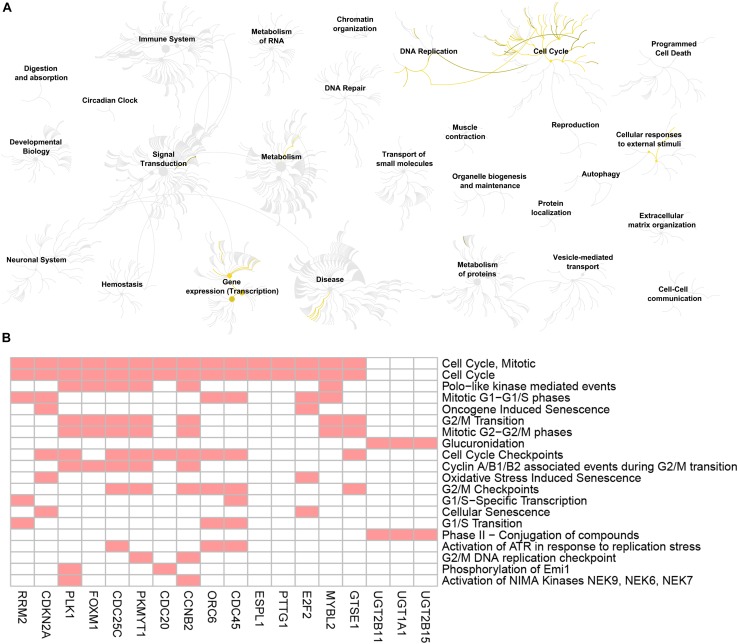
Reactome pathway analysis of the genes screened by GO biological process analysis. **(A)** Visualization of genome-scale Reactome pathway analysis results of the 19 genes screened by GO biological process analysis, showing an intuitive overview of analysis results in the context of the Reactome hierarchical pathway structure. Enriched pathways are plotted in yellow. **(B)** Heatmap of the genes enriched in the top 20 significant Reactome pathways. Red boxes mean that the corresponding gene (X-axis) is enriched in the corresponding Reactome pathway (Y-axis).

To reduce data dimensionality, three types of enrichment analyses were utilized to obtain the most prominent genes in the black module. As mentioned above, KEGG pathway enrichment analysis showed several pathways that were correlated with oncogenesis. The KEGG pathway is a collection of manually drawn pathway maps representing current knowledge on the molecular interaction and reaction networks and interpreting the data in the context of biological processes, pathways and networks. Twelve pathways were significantly enriched (Figure 4A), and among these pathways, five pathways including cell cycle, cellular senescence, p53 signaling pathway, steroid hormone biosynthesis and pentose and glucuronate interconversions are widely reported to be correlated with the development and progression of several types of cancers (Kastan and Bartek, 2004; Lange et al., 2007; Munoz-Fontela et al., 2016; Calcinotto et al., 2019; Liu et al., 2019). In total, 23 DEGs were enriched in the five pathways; we selected all 23 genes (Figure 4B) and performed GO enrichment analysis. Many processes were significantly enriched and the top 20 significant processes were extracted for further investigation (Figure 5A). Likewise, 19 DEGs were enriched in all 20 processes and all 19 genes were selected (Figure 5B) and used for next-step enrichment analysis. REACTOME is a pathway database that provides intuitive bioinformatics tools for the visualization, interpretation and analysis of pathway knowledge (Fabregat et al., 2018). Here, REACTOME pathway enrichment analysis was also conducted using the REACTOME pathway analysis tool (Fabregat et al., 2017), and pathways such as “Cell Cycle Checkpoints” were reportedly correlated with several cancer types (Figure 6A) (Kastan and Bartek, 2004; Malumbres and Barbacid, 2009). Literature review was performed on these pathways, and 13 genes (*RRM2, ORC6, CDC45, CDKN2A, E2F2, MYBL2, CCNB2, PLK1, FOXM1, CDC25C, PKMYT1, GTSE1, and CDC20*) in nine correlated pathways (mitotic G1-G1/S phases, oncogene induced senescence, G2/M transition, mitotic G2-G2/M phases, cell cycle checkpoints, G2/M checkpoints, G1/S-specific transcription, G1/S transition, and G2/M DNA replication checkpoint) were finally identified and speculated to be potentially correlated with prostate cancer (Figure 6B) (Nakayama and Nakayama, 2006; Malumbres and Barbacid, 2009).

### WGCNA of miRNAs

**FIGURE 7:**
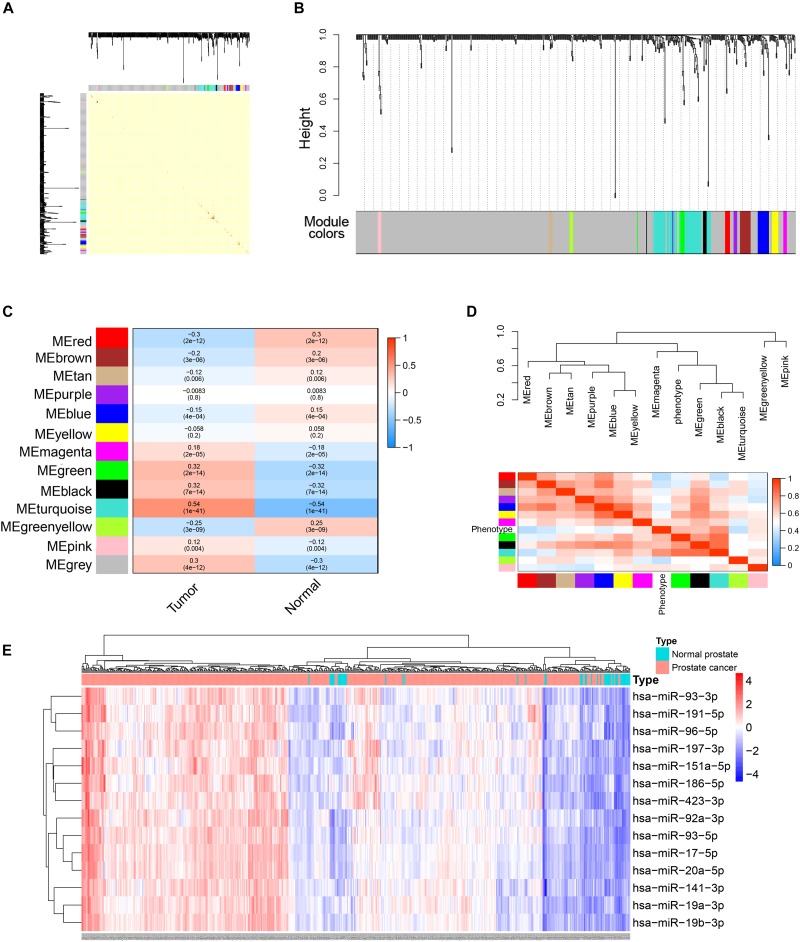
WGCNA analysis of miRNAs expression data in TCGA PRAD datasets. **(A)** Topological overlap matrix plot showing topological overlap in the gene network of miRNAs. **(B)** Clustering dendrograms for the miRNAs with dissimilarity based on the topological overlap together with the assigned module colors. **(C)** Module-trait relationships. Each cell contains the corresponding correlation coefficient and *p*-value. **(D)** The eigengene dendrogram and heatmap plot of the adjacencies of modules. Red represents high adjacency (positive correlation) and blue represents low adjacency (negative correlation). **(E)** Heatmap and cluster analysis (Euclidean distance) of the 14 hub miRNAs obtained by WGCNA analysis of miRNAs. Rows correspond to miRNAs and columns correspond to samples. Red and blue colors indicate different extents of high or low expressions.

To better understand the roles of miRNAs in prostate cancer, we further performed WGCNA analysis for miRNAs based on the expression data obtained from TCGA database. Because the total count of miRNA was far fewer than that of genes, differential expression analysis was not conducted before WGCNA. Similar to the analysis pipeline of WGCNA for genes, a one-step network was constructed (Figures 7A,B) and correlations between trait and modules as well as intramodular correlations were calculated (Figures 7C,D). The turquoise module had the highest correlation coefficient. The connectivity of each miRNA with other miRNAs was calculated and 14 hub miRNAs (miRNAs with the highest module membership) were selected according to the following criteria: miRNA significance value (calculated by WGCNA and representing the correlation between miRNA expression and trait) > 0.3; and absolute value of module membership (calculated by WGCNA and representing the degree each miRNA is associated with the module) > 0.8 (Figure 7E). Based on this analysis, these miRNAs were very likely to interact with target genes and function in the development of prostate cancer.

### Construction of Gene–miRNA Interaction Network and Analysis of Hub Genes and miRNAs

**FIGURE 8:**
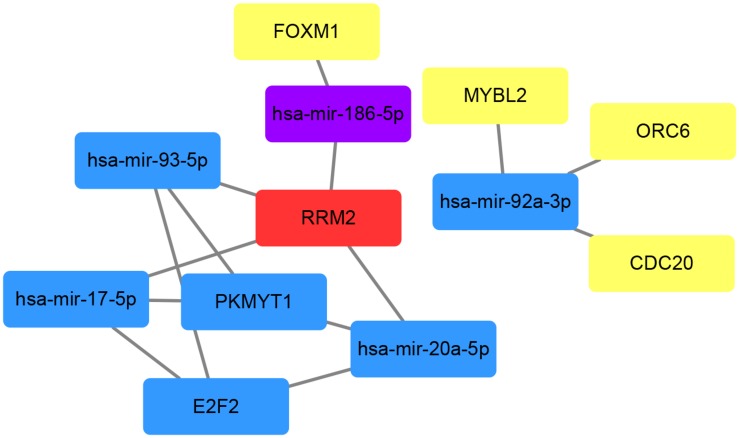
Schematic plot of the gene–miRNA interactions based on miRNet. MiRNet catalogues predicted and validated miRNA–gene interactions. MiRNAs targeting at least two genes were selected to find intersections with the hub miRNAs obtained by WGCNA analysis. Red represents four interactions with other genes or miRNAs in the network, while blue for 3, purple for 2, and yellow for 1.

To investigate the interactions between the screened miRNAs and genes, we used the miRNet tool which contains miRNA–gene interaction data collected from miRTarBase v7.0, TarBase v7.0, and miRecords and determined the miRNAs that were predicted or validated to target the 13 genes obtained after pathway enrichment analyses. Among all 339 miRNAs, 66 targeted at least two DEGs and were used to find intersections with the 14 hub miRNAs. Five miRNAs (*hsa-mir-17-5p, hsa-mir-20a-5p, hsa-mir-92a-3p, hsa-mir-93-5p, and hsa-mir-186-5p*) were commonly shared. Then the interactions between the 5 miRNAs and the aforementioned 13 DEGs were extracted from the miRNA–gene interaction data analyzed using the miRNet tool. According to miRNet, 7 genes (*RRM2, ORC6, E2F2, FOXM1, PKMYT1, CDC20, and MYBL2*) among the 13 DEGs had interactions with the 5 miRNAs. The gene–miRNA interactions of the five miRNAs and seven genes were plotted using Cytoscape software (Figure 8). Three genes (*E2F2, RRM2, and PKMYT1*) had three or more interactions with miRNAs and four miRNAs (*hsa-mir-17-5p, hsa-mir-20a-5p, hsa-mir-92a-3p, and hsa-mir-93-5p*) had interactions with three genes. Given the high connectivity of the three genes and four miRNAs, it is reasonable to presume that they might be a potential signature in prostate cancer.

### Investigation on the Clinical Prognostic Significance of Signature Genes and Expression of Signature miRNAs in Gene Expression Omnibus Datasets

**FIGURE 9:**
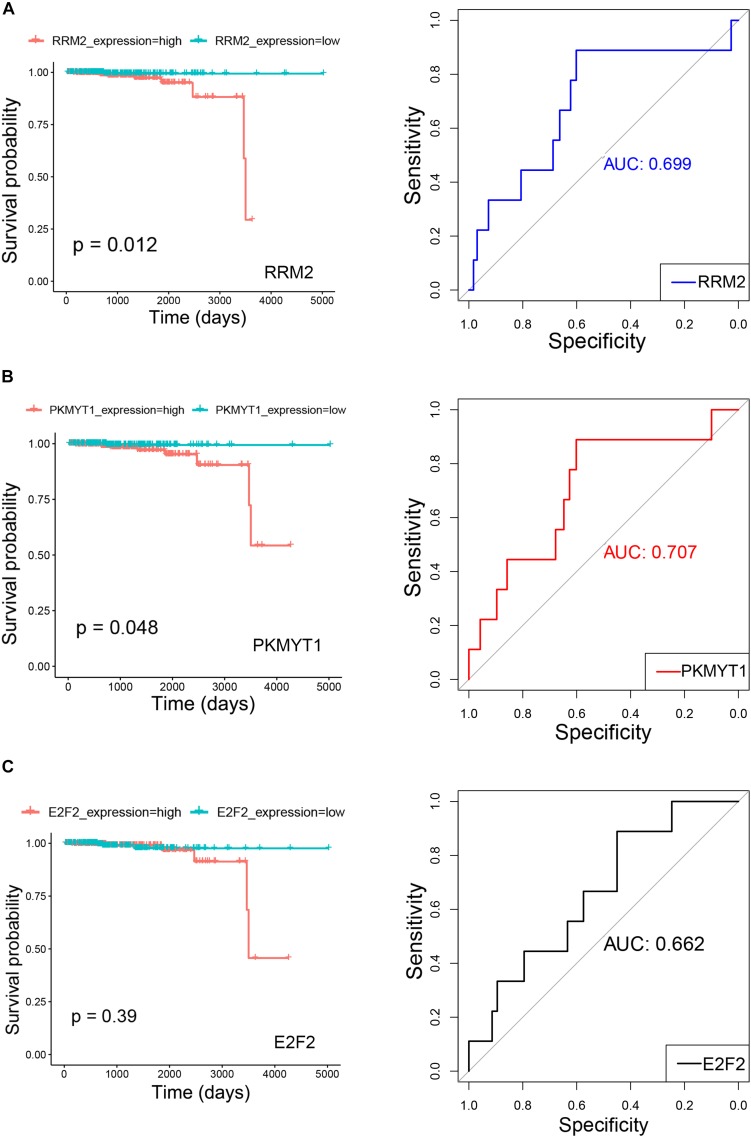
Kaplan–Meier survival curves and receiver operating characteristic (ROC) curves showing correlations of RRM2 **(A)**, PKMYT1 **(B)**, and E2F2 **(C)** with clinical survival (TCGA PRAD survival data). In Kaplan–Meier survival curves, red curves refer to high expression group (greater than median) and green curves refer to low expression group (equal to or smaller than median). Both *p*-values for Kaplan–Meier survival curves and AUC (Area under the ROC Curve) values for ROC curves are plotted.

**FIGURE 10:**
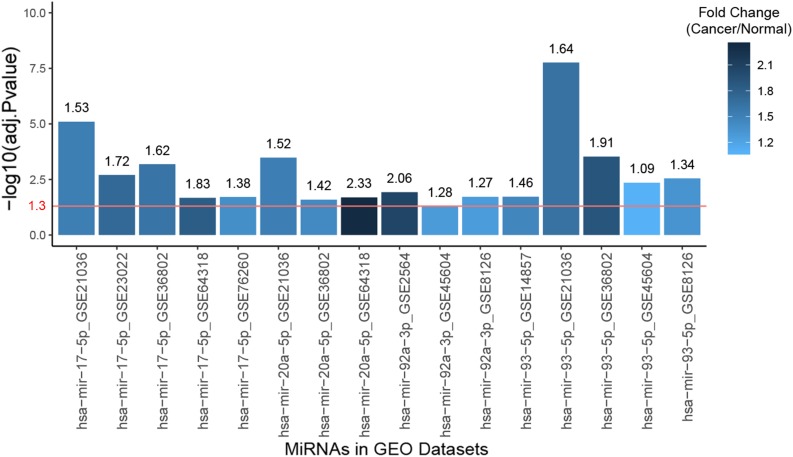
Bar plot of the expression fold changes of the four hub miRNAs in prostate cancer compared to normal prostate tissues according to several studies in Gene Expression Omnibus (GEO) datasets. X-axis correspond to miRNA names and GEO study accessions while Y-axis correspond to negative log10-transformed *p*-values. The red line indicates 1.3 in Y-axis, which is the negative log10-transformed value for 0.05, meaning the fold changes of the bars higher than this line are statistically significant.

The clinical prognostic significance was studied for *RRM2*, *PKMYT1*, and *E2F2*. According to Kaplan–Meier survival analyses and survival ROC curve analyses, higher expression of *RRM2* or *PKMYT1* was significantly correlated with worse survival (*p* < 0.05, AUC > 0.69) (Figures 9A,B). For *E2F2*, higher expression did not indicate worse survival but ROC curve still showed considerable AUC value (Figure 9C). To conduct some validation of sorts, we further studied the expression of four signature miRNAs in several Gene Expression Omnibus (GEO) datasets. The GEO database is an independent public repository that archives and freely distributes high-throughput gene expression and other functional genomics datasets (Barrett et al., 2013). All four miRNAs were more highly expressed in prostate cancer than in normal prostate tissues and this was confirmed by at least three GEO datasets for each miRNA (Figure 10). Among the four miRNAs, three members (*hsa-mir-17-5p, hsa-mir-20a-5p, and hsa-mir-93-5p*) had some of the strongest *p*-values for tumor and normal differential expression in the independent GEO datasets (GSE21036, and GSE36802). All of these data provide strong evidence that the signatures of three genes and four miRNAs play crucial roles in the development of prostate cancer.

## Discussion

With the increasing number of studies focusing on prostate cancer, much progress has been made in the treatment and understanding of the mechanisms underlying this disease. However, controversies remain about several issues such as the screening for prostate cancer and the treatment of localized disease (Pignot et al., 2018). The optimum management of prostate cancer is challenging partly due to the heterogeneity and sometimes indolent nature of the disease. Detailed biology of oncogenesis and progression of prostate cancer, especially advanced subtypes, still needs to be elucidated for screening, stratification and treatment strategies. Thus, a signature with prognostic and therapeutic implications is of great significance.

Integrative analysis of accumulated expression data can be performed to obtain more reliable information and more feasible measures for determining the potential diagnostic and prognostic signatures of tumors and to investigate the molecular mechanisms (Robertson et al., 2017; Li et al., 2018; Ye et al., 2018). WGCNA builds network modeling that relies on statistical methods and improves simple correlation networks by quantifying not only the correlations between individual pairs of genes, but also the extent to which these genes share the same neighbors (Zhang and Horvath, 2005; DiLeo et al., 2011). WGCNA provides an efficient approach through which the effects of phenotype can be detected in modeled networks (Langfelder and Horvath, 2008). Many studies have indicated that cancer biology is regulated by miRNAs. Research works focusing on miRNA-based cancer treatments are gaining increasing attention due to the ability of miRNAs to concurrently target multiple effectors of pathways involved in cell differentiation, proliferation, and survival (Garzon et al., 2010). In this study, using the expression data of genes and miRNAs of prostate cancer in TCGA database as well as the WGCNA approach, we identified three genes and four miRNAs that were tightly correlated with prostate cancer and clinical prognosis.

In our pathway enrichment analyses, we utilized three databases, namely KEGG, GO, and REACTOME. For each kind of functional enrichment analysis, there were always several pathways relevant to the cell cycle that were enriched. It is universally accepted that dysregulation of the cell cycle is characteristic of tumors, which further proved that our functional enrichment analyses were properly conducted (Malumbres and Barbacid, 2009).

Protein kinase membrane-associated tyrosine/threonine 1 (*PKMYT1*) inhibits progression of the cell cycle through the phosphorylation of cyclin-dependent kinase 1 (*CDK1*) (Choi et al., 2009). It is well-established that *CDK1* is a key molecule in the progression of mitosis, and thus *PKMYT1* is thought to play important roles in the regulation of the cell cycle and tumor biology (Parker and Piwnica-Worms, 1992). *PKMYT1* is a crucial promoter in the development of hepatocellular carcinoma (Aguiar et al., 2017; Liu et al., 2017), and overexpression of *PKMYT1* indicates a poor prognosis and enhances proliferation and tumorigenesis in non-small cell lung cancer (Sun et al., 2019). Currently the functional significance of *PKMYT1* in prostate cancer remains unclear, and it makes sense to determine whether *PKYMT1* is indispensable in prostate cancer, which requires further investigation. The ribonucleotide reductase M2 subunit (*RRM2*) plays important role in several human cancers, including colorectal cancer, hepatocellular carcinoma, pancreatic adenocarcinoma, and breast cancer (Lee et al., 2014; Sierzega et al., 2017; Nana et al., 2018; Chen et al., 2019). Increased expression of *RRM2* has been demonstrated to be a mechanism driving poor patient outcomes in prostate cancer, in accordance with our findings (Mazzu et al., 2019). According to our study, the high expression of *PKMYT1* and *RRM2* was correlated with prostate cancer oncogenesis and poor prognosis, indicating their potential oncogenic roles in prostate cancer. *E2F* transcription factor 2 (E2F2) is one of the activators of the family that controls the transition between the G1 and S phase through various upstream signals (Sherr, 1996), and functions as an oncogene in lung cancer (Feliciano et al., 2017). Li et al. (2017), reported that *E2F2* variants are predictive biomarkers for recurrence risk in patients with squamous cell carcinoma of the oropharynx. It is documented that downregulated *E2F2* is correlated with inhibition of cell proliferation in prostate cancer (Dong et al., 2010). However, more research regarding its roles in prostate cancer is still needed. Among the four hub miRNAs found in this study, *miRNA-17-5p* has been extensively investigated (Hussein et al., 2009; Dellago et al., 2017). *MiRNA-17-5p* is shown by multiple studies to be at the crossroads of aging and cancer and might be a promising biomarker or even therapeutic tool and target in tumors (Dellago et al., 2017). The high expression of *miRNA-17-5p* is significantly associated with the progression of various cancers probably through increasing the expression of *p53* (Thompson et al., 2016; Duan et al., 2018). However, *miRNA-17-5p* also reportedly acts as a tumor suppressor in triple-negative breast cancer, which is the breast cancer subtype with the poorest prognosis (Wang et al., 2019). Aberrant expression of *miRNA-92a* has been reported in various tumors and as such has potential value as a tumor marker or novel target for cancer treatment (Li et al., 2014). In prostate cancer, the expression of *miR-92a* is markedly overexpressed according to a large-scale miRnome analysis (Volinia et al., 2006). For each gene or miRNA included in the signature found in this study, our report provided novel targets, or at least presented further confirmation of previously published findings of prostate cancer.

It is worth noting that all exploratory data analysis approaches such as WGCNA require further validation to confirm the putative molecular networks. In addition, the expression of a specific gene or miRNA might vary among different subtypes of prostate cancer, especially taking into account the heterogeneous nature of this disease (Boyd et al., 2012).

A signature with prognostic significance makes great sense for prostate cancer patients. For instance, a signature identifying prostate cancer patients at risk of dying and who can be safely observed is of tremendous importance, as we are aware of overtreatment concerns and more patients are being placed on active surveillance protocols. This study preliminarily investigated potential candidate drivers in prostate cancer as well as the underlying mRNA–miRNA interactions, providing potential therapeutic targets for prostate cancer. It will be of great interest to determine whether this signature will prove useful and gain traction in further studies.

## Data Availability Statement

All datasets generated for this study are included in the article/supplementary files.

## Author Contributions

JW, YY, QD, JZ, YW, GY, JY, and YT: conception. JW, YY, QD, JZ, and YW: data collection and analyses. JW, YY, and QD: writing, review, and/or revision of the manuscript. GY, JY, and YT: study supervision.

## Conflict of Interest

The authors declare that the research was conducted in the absence of any commercial or financial relationships that could be construed as a potential conflict of interest.

## Footnotes

1 https://xena.ucsc.edu

2 https://biit.cs.ut.ee/gprofiler

3 https://reactome.org

4 https://www.mirnet.ca

5 http://www.picb.ac.cn/dbDEMC

6 https://www.r-project.org

7 https://rstudio.com
